# Ang–Tie Angiogenic Pathway Is Distinctively Expressed in Benign and Malignant Adrenocortical Tumors

**DOI:** 10.3390/ijms23105579

**Published:** 2022-05-17

**Authors:** Sofia Oliveira, Sofia S. Pereira, Madalena M. Costa, Mariana P. Monteiro, Duarte Pignatelli

**Affiliations:** 1 UMIB–Unit for Multidisciplinary Research in Biomedicine, ICBAS–Instituto de Ciências Biomédicas Abel Salazar, 4050-313 Porto, Portugal; up201909110@fc.up.pt (S.O.); mcosta@icbas.up.pt (M.M.C.); mpmonteiro@icbas.up.pt (M.P.M.); 2 ITR–Laboratory for Integrative and Translational Research in Population Health, 4050-600 Porto, Portugal; 3 Instituto de Investigação e Inovação em Saúde (I3S), Universidade do Porto, 4200-135 Porto, Portugal; dpignatelli@ipatimup.pt; 4 Institute of Molecular Pathology and Immunology (IPATIMUP), University of Porto, 4200-135 Porto, Portugal; 5 Department of Endocrinology, Hospital S. João, 4200-319 Porto, Portugal; 6 Department of Biomedicine, Faculty of Medicine, University of Porto, 4200-319 Porto, Portugal

**Keywords:** adrenocortical tumors, diagnosis, prognosis, angiogenesis, VEGF pathway, Ang–Tie pathway

## Abstract

The differential diagnosis between adrenocortical adenomas (ACAs) and adrenocortical carcinomas (ACCs) relies on unspecific clinical, imaging and histological features, and, so far, no single molecular biomarker has proved to improve diagnostic accuracy. Similarly, prognostic factors have an insufficient capacity to predict the heterogeneity of ACC clinical outcomes, which consequently lead to inadequate treatment strategies. Angiogenesis is a biological process regulated by multiple signaling pathways, including VEGF and the Ang–Tie pathway. Many studies have stressed the importance of angiogenesis in cancer development and metastasis. In the present study, we evaluated the expression of VEGF and Ang–Tie pathway mediators in adrenocortical tumors (ACTs), with the ultimate goal of assessing whether these molecules could be useful biomarkers to improve diagnostic accuracy and/or prognosis prediction in ACC. The expression of the proteins involved in angiogenesis, namely CD34, VEGF, VEGF-R2, Ang1, Ang2, Tie1 and Tie2, was assessed by immunohistochemistry in ACC (*n* = 22), ACA with Cushing syndrome (*n* = 8) and non-functioning ACA (*n* = 13). ACC presented a significantly higher Ang1 and Ang2 expression when compared to ACA. Tie1 expression was higher in ACC with venous invasion and in patients with shorter overall survival. In conclusion, although none of these biomarkers showed to be useful for ACT diagnosis, the Ang–Tie pathway is active in ACT and may play a role in regulating ACT angiogenesis.

## 1. Introduction

Adrenocortical tumors (ACTs) originate in the adrenal cortex and affect 3 to 10% of the human population [1]. A majority of ACTs have a benign behavior, are hormonally non-functioning and are most often diagnosed incidentally in the course of imaging examinations performed for unrelated health conditions [2]. In contrast to adrenocortical adenomas (ACAs), adrenocortical carcinomas (ACCs) are rare and usually very aggressive tumors. The differential diagnosis between ACC and ACA, despite being driven by a few clinical and imaging characteristics, relies mainly on the histopathologic criteria that comprise the Weiss system and Ki-67 expression [3]. However, tumor morphological features are poorly specific, and there can be considerable overlap between ACA and ACC that contribute to diagnostic inaccuracy. Although ACCs often present a poor prognosis, individual variability in tumor progression and survival is well recognized. Besides tumor stage, disease heterogeneity seems to be related to different biological and molecular profiles within ACC, reinforcing the unmet need to identify novel biomarkers with added prognostic value [4,5].

Angiogenesis represents the growth and development of new blood vessels from pre-existing vasculature, an important phenomenon in tumor biology which is involved in tumor progression [6]. Similar to other complex biological functions, several signaling pathways are involved in the initiation, growth and maintenance of blood vessels, including the vascular endothelial growth factor (VEGF) and Ang–Tie pathways [7].

VEGF is the most potent inductor of angiogenesis. VEGF activates the vascular endothelial receptor 2 (VEGF-R2) that, in turn, induces angiogenesis by promoting proliferation, migration and cell survival. The Ang–Tie signaling cascade includes angiopoietin 1 (Ang1) and 2 (Ang2) and the transmembrane receptors Tie1 and Tie2. This pathway seems to act as VEGF co-adjuvant by controlling later stages of angiogenesis, while regulating vascular permeability and remodeling. The VEGF status of adrenocortical tumors was previously described in Reference [8]. However, despite having an important role on fetal adrenal gland angiogenesis, the status of the Ang–Tie pathway signaling in ACT has not been previously reported [8].

In the present work, we aimed to evaluate the expression of molecules that participate in VEGF and Ang–Tie pathways in ACT and its potential use for fine-tuning differential diagnosis and/or prediction of ACC prognosis.

## 2. Results

### 2.1. Angiogenic Proteins Expression in ACT

#### 2.1.1. CD34 Expression

**Figure 1:**
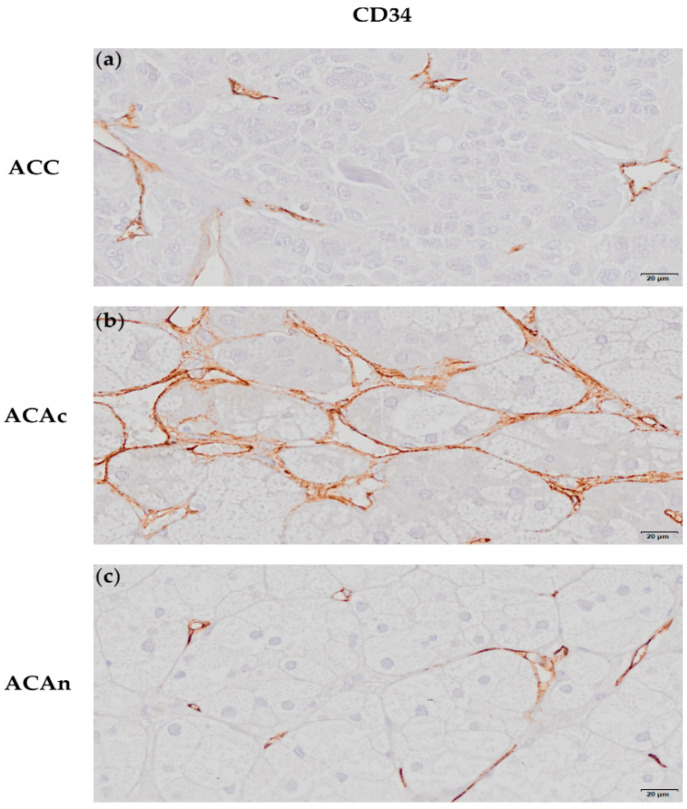
Immunochemistry staining of an adrenocortical carcinoma (ACC) (**a**), adrenocortical adenoma with Cushing syndrome (ACAc) (**b**) and non-functioning adrenocortical adenoma (ACAn) (**c**) for CD34 (20 µm). CD34 positively stains the endothelial cells (**a**–**c**).

CD34 immunochemistry (IHC) staining was used to evaluate ACT tumor blood vessel density (Figure 1a–c).

**Figure 2:**
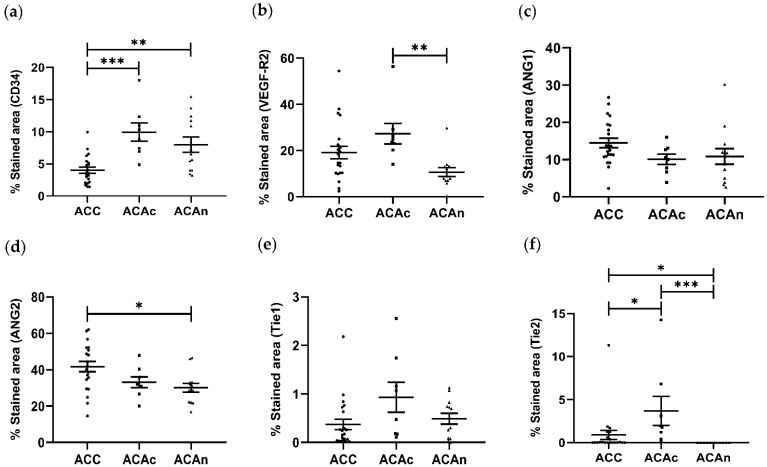
Distribution of the stained area for CD34 (**a**), VEGF-R2 (**b**), Ang1 (**c**), Ang2 (**d**), Tie1 (**e**) and Tie2 (**f**) in adrenocortical carcinoma (ACC), adrenocortical adenoma with Cushing syndrome (ACAc) and non-functioning adrenocortical adenoma (ACAn). (ANOVA or Kruskal–Wallis test: * *p* < 0.05, ** *p* < 0.01 and *** *p* < 0.001).

The percentage of CD34 staining area was significantly lower in ACC (4.023 ± 0.408%) when compared to both adrenocortical adenoma with Cushing syndrome (ACAc) (9.947 ± 1.431%, *p* < 0.01) and non-functioning adrenocortical adenoma (ACAn) (7.988 ± 1.188%, *p* < 0.001). No differences were observed between ACAn and ACAc (Figure 2a).

#### 2.1.2. VEGF and VEGF-R2 Expression

**Figure 3:**
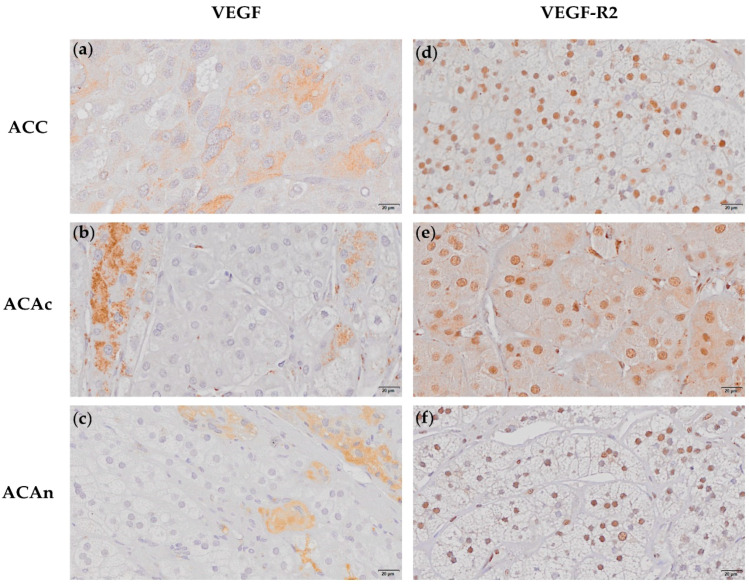
Immunochemistry staining of adrenocortical carcinoma (ACC), adrenocortical adenoma with Cushing syndrome (ACAc) and non-functioning adrenocortical adenoma (ACAn) for VEGF (20 µm) (**a**–**c**) and VEGF-R2 (20 µm) (**d**–**f**). Different VEGF immunochemistry patterns presenting cytoplasm staining (**a**–**c**); VEGF-R2 nuclear staining in ACC, ACAc and ACAn (**d**–**f**).

All ACTs expressed VEGF with a variable expression pattern within the tumor cells. VEGF expression was found in the cytoplasm and nucleus in 38% of ACC and ACAn (Figure 3a–c and Supplementary Figure S1). VEGF expression was exclusively found in the cytoplasm in the remaining ACC, ACAn and in all ACAc (Figure 3a–c). However, there was no significant difference in the VEGF expression pattern when the ACT groups were compared (*p* = 0.09) (Table 1).

VEGF-R2 expression was also present in every ACT. In addition, to cytoplasmatic expression, VEGF-R2 nuclear expression was also observed in all ACC, ACAc and ACAn (Figure 3d–f). However, the percentage of the VEGF-R2 stained area was significantly lower in ACAn (10.639 ± 1.900%) than in ACAc (27.298 ± 4.454%, *p* < 0.05) (Figure 2b). No differences were observed when comparing ACC and ACA.

#### 2.1.3. Ang1 and Ang2 Expression

**Figure 4:**
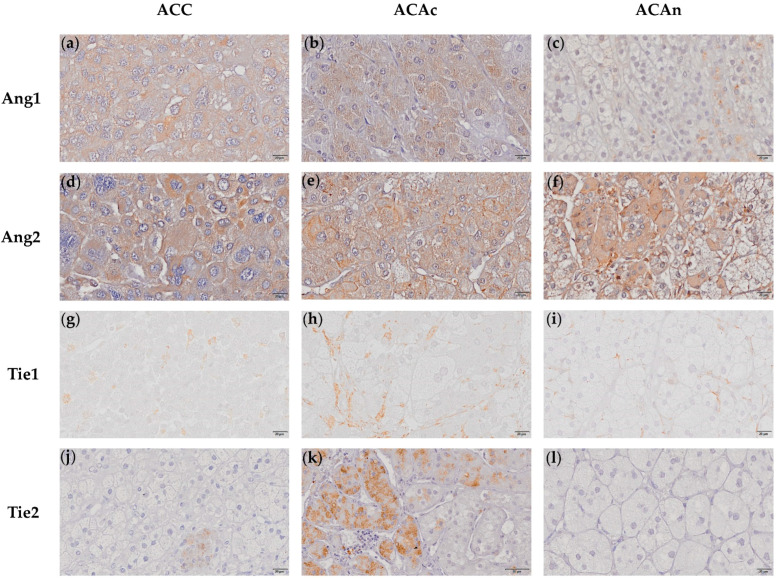
Immunochemistry staining of adrenocortical carcinoma (ACC), adrenocortical adenoma with Cushing syndrome (ACAc) and non-functioning adrenocortical adenoma (ACAn) for Ang1 (20 µm) (**a**–**c**), Ang2 (20 µm) (**d**–**f**), Tie1 (20 µm) (**g**–**i**) and Tie2 (20 µm) (**j**–**l**). Cytoplasm staining for Ang1 in ACC, ACAc and ACAn (**a**–**c**); Ang2 cytoplasm staining (**d**–**f**); Tie1 cytoplasm staining (**g**–**i**); Tie2 cytoplasm staining in ACC and ACAC (**j**–**k**); and negative staining of Tie2 in ACAn cells (**l**).

Ang1 was found to be expressed in all ACTs (Figure 4a–c). ACC (14.479 ± 1.279%) presented a numerically greater Ang1 stained area when compared to ACAc (10.087 ± 1.377%) and ACAn (10.843 ± 2.088%), with no significant differences between groups (Figure 2c).

Ang2 positive staining for was observed in all ACTs (Figure 4d–f). The Ang2 stained area was significantly higher in ACC when compared to ACAn (41.731 ± 2.832% vs. 30.097 ± 2.428%, *p* < 0.05) (Figure 2d).

#### 2.1.4. Tie1 and Tie 2 Expression

Tie1 expression was present in all ACTs, except in a single ACAn (Figure 4g–i). No significant differences were observed between ACC and both ACAc and ACAn (ACC, 0.373 ± 0.107%; ACAc, 0.933 ± 0.310%; and ACAn, 0.489 ± 0.111%) (Figure 2e).

Tie2 expression was present in all ACAc and in 57.1% of ACC. In contrast, Tie2 staining was negative in ACAn and in 42.9% of ACC (Figure 4j–l). The percentage of Tie2 stained area was significantly higher in ACAc when compared to ACC (3.695 ± 1.682% vs. 0.881 ± 0.539%, *p* < 0.05) (Figure 2f). In addition to Tie expression, both Tie1 and Tie2 receptors presented cytoplasm localization in all ACT with positive staining.

### 2.2. Correlation between Angiogenic Biomarkers and Patients and Tumor Characteristic

No significant correlations were found between the angiogenic markers in ACC, ACA and tumor or patient characteristics (Supplementary Tables S1–S3).

Within ACC, the percentage of stained area for each protein was compared across patient subgroups according to several tumor features, including European Network for the Study of Adrenal Tumors (ENSAT) Stage; and presence or absence of distant metastasis, capsule, venous and sinusoids invasion (Table 2). A higher Tie1 expression was found in ACC with venous invasion when compared to ACC without venous invasion (0.431 ± 0.133% vs. 0.099 ± 0.031%, *p* = 0.021) (Table 2). No additional differences were observed between any other angiogenic markers, including VEGF expression pattern, and the ACC parameters analyzed (Supplementary Table S4).

### 2.3. Angiogenic Markers Accuracy for Differential Diagnosis

**Figure 5:**
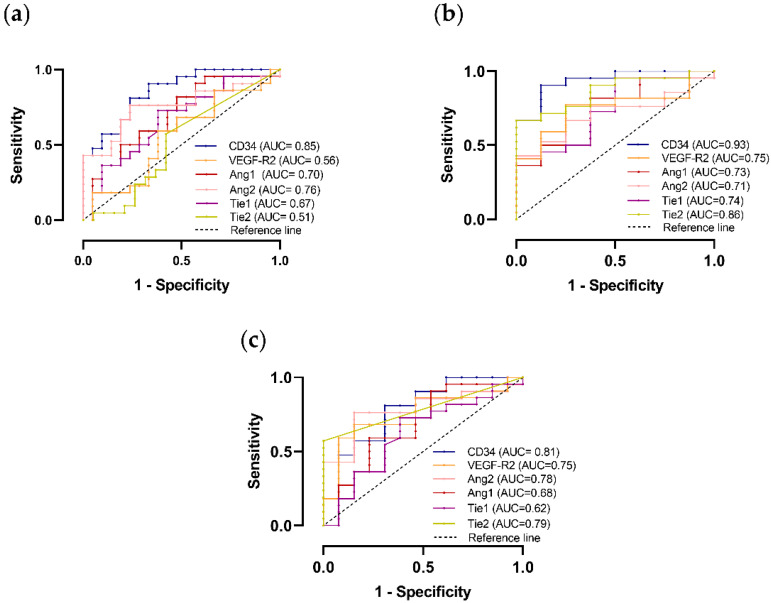
Graphic representation of ROC curve to distinguish adrenocortical carcinoma (ACC) and total adrenocortical adenomas (ACAt) (**a**); ACC and adrenocortical adenoma with Cushing syndrome (ACAc) (**b**); ACC and non-functioning adrenocortical adenoma (ACAn) (**c**) with the respective area under the curve (AUC).

CD34 was demonstrated to have good accuracy for the differential diagnosis between ACC and ACA on ROC curve analysis. This molecular marker was particularly accurate in differentiating ACC and functioning ACA (ACAc). In addition, the Tie2 receptor also proved to be a good marker in distinguishing ACC from ACAc (Figure 5b).

Despite the differences observed in molecular expression when comparing ACA and ACC, the remaining biomarkers analyzed presented low accuracy in regard to distinguishing ACC from ACA (Figure 5 and Supplementary Figure S2).

### 2.4. Angiogenic Markers Accuracy to Predict ACC Prognosis

There was no significant difference in angiogenic markers expression nor VEGF expression pattern in ACC according to patient overall survival. A higher Tie1 expression was found in most patients with lower survival (Table 3).

## 3. Discussion

The poor specificity of pathologic findings used to diagnose ACC and predict disease prognosis are major contributors to diagnosis inaccuracy and inappropriate treatment interventions. In the present study, the expression of VEGF and Ang–Tie pathways’ molecular intermediates of ACT was characterized, while the potential for increasing the diagnosis and/or prognosis prediction accuracy was evaluated.

In our study, CD34 expression was used to assess vascular density. Although a high vascular density was globally present in ACT, this was significantly lower in ACC when compared to ACA.

Previous studies on ACC and ACA vascular density yielded inconsistent results [9,10,11,12,13,14]. This could be attributed to the use of different methods to quantify vessels’ density or tumor functionality bias, which are known to influence angiogenesis. Although CD34 expression was demonstrated to be an excellent biomarker to distinguish ACC from ACAc, in the present study, this is more likely related to steroid production capacity than with other tumor biological features. In line with that, Cox regression demonstrated that CD34 expression in ACC is not related to patients’ overall survival.

In addition to vascular density analysis, the status of VEGF and Ang–Tie pathways in ACT was assessed, thus enabling us to demonstrate that both VEGF and Ang–Tie pathways are expressed in ACT.

In ACC cells, VEGF expression can depict two different location patterns, in cytoplasm only or in both the nucleus and cytoplasm. Nuclear expression was found in 38% of ACC and of ACAn, whereas, in the remaining tumors, immunostaining was exclusively present in the cytoplasm. VEGF nuclear expression in other tumor types, such as breast cancer and colorectal cancer, has been previously reported [15,16]. However, to the best of our knowledge, VEGF nuclear expression in ACT is herein first described. Nevertheless, the different VEGF expression patterns were not invariably associated with malignancy, since VEGF nuclear expression was not exclusively observed in ACC, and there were no significant differences between groups.

In our study, ACAc presented a significantly greater VEGF-R2 expression when compared to ACAn. VEGF is highly expressed by the adrenal cortex and required to maintain a dense and fenestrated vasculature necessary for efficient steroid secretion [17]. Therefore, a higher VEGF-R2 expression found in ACAc is unsurprising and could be related to the tumor hormone secretion profile. Moreover, in addition to the expected VEGF-R2 expression in the cytoplasm, nuclear expression was also observed in all ACTs. Indeed, VEGF-R2 nuclear expression has been previously described in other pathological conditions and seems to be associated with tumor cell proliferative capacity, suggesting that nuclear expression may be involved in the mechanisms that contribute for tumor progression [18]. Thus, the VEGF-R2 nuclear location in ACT could contribute to angiogenic response amplification, a phenomenon that is not exclusive of malignant tumors. Despite the differences in the molecular expression pattern identified, VEGF and VEGF-R2 failed to demonstrate any diagnostic or prognostic value in ACC patients.

This study is also the first to report that the Ang–Tie pathway signaling molecules are expressed in ACT. Ang2 can play different roles in tumor angiogenesis. In the presence of VEGF, Ang2 acts synergistically with VEGF to promote angiogenesis, while, in the absence of VEGF, Ang2 induces blood vessel regression [19]. Since VEGF expression was identified in every ACT, this finding suggests that Ang2 is more likely to promote angiogenesis and vascular permeability. Moreover, Ang2 expression was higher in ACC than in ACA, which further supports the hypothesis of Ang2 being associated with greater vascular permeability. Thus, although ACC presents a lower vessel density, our study suggests that these vessels are likely to be more permeable, thus contributing to tumor cell dissemination.

ACC with venous invasion also presented a greater Tie1 expression when compared to ACC without venous invasion. This finding might be potentially explained by endothelial wall disruption, which results in loss of the venous endothelium quiescent state, which, in turn, triggers Tie1 expression [20]. In addition, higher Tie1 expression was associated with lower patient survival that could be potentially linked to the rate of ACC progression associated with venous invasion. In benign ACT, Tie2 was only expressed in ACAc. Moreover, 42.9% of ACC expressed Tie2, although the expression was lower than in ACAc. Taken together, these results suggest that Tie2 expression might be related to tumor functionality. The Tie2 receptor is the main mediator of Ang1 and Ang2 biological functions, in such a way that, at higher expression levels, Ang2 binds and activates Tie2 [21]. Since Ang2:Ang1 in ACT was in favor of Ang2, Tie2 activation could be the end result of Ang2 binding.

Despite the differences observed, neither angiopoietins nor tie receptors demonstrated to be useful biomarkers for the differential diagnosis of ACT on ROC curve analyses.

## 4. Materials and Methods

### 4.1. Case Selection

Adrenal tissue was obtained during adrenalectomy from patients with ACTs (*n* = 43), including ACC (*n* = 22), ACAc (*n* = 8) and ACAn (*n* = 13). A summary of patients’ and tumors’ characteristic is given in Table 4.

### 4.2. Immunochemistry and Data Analysis

IHC was performed in 3 µm formalin-fixed paraffin embedded tissue sections mounted on adhesive microscope slides (Superfrost^®^ Plus, Thermo Fisher Scientific, Waltham, MA, USA). Tissue sections were deparaffinized in xylene and hydrated in downgraded alcohols before undergoing antigen retrieval, as described in Table 5. After that, sections were rinsed in the respective washing solution (Table 5), followed by a treatment with 3% hydrogen peroxide (H_2_O_2_) (MERCK, Darmstadt, Germany) in methanol to inhibit endogenous peroxidase for 15 min. For Ang2, tissue sections were also incubated with 10% bovine serum albumin (BSA) at room temperature for 30 min to block non-specific reactions. Then tissue sections were incubated overnight at 4 °C with the primary antibody. The detection of the immune reaction was performed by incubation for 60 min with the commercial Dako Real™ EnVision™ Detection System (K5007, Dako, Næstved, Denmark). Then 3,3′-diaminobenzidine (DAB) was used as chromogen, and hematoxylin as nuclear counterstaining.

Slides were scanned for each marker, using the image-acquisition Olympus^®^ VS110™ virtual slide scanning system and captured by using the image-acquisition software VS-ASW (v 2.3 for Windows). The tumor area was delimited by a pathologist, and adjacent tissue and capsule were not included in the analysis. IHC images were analyzed by using the software ImageJ (National Institutes of Health, Bethesda, MD, USA) that allows for the detachment of the stained area from each histological image based on the Red–Green–Blue (RGB) system. The percentage of the stained area for each protein was calculated through the ratio between the stained and the total tissue area. Unspecific staining that included areas of necrosis and fibrosis was excluded. VEGF expression presented two different patterns, with cell staining occurring both in the cytoplasm and nucleus, or in cytoplasm only. Thus, VEGF expression pattern evaluation was first identified by direct observation and then quantified in tumors that presented cytoplasm and nuclear expression and cytoplasm expression only.

### 4.3. Statistical Analysis

All ordinal data are represented as mean ± standard error of the mean (SEM). To evaluate the variables’ normality, the D’Agostinho–Pearson test was used. For variables that passed this test, the one-way ANOVA test with the post hoc Tukey was used to compare the means of 3 groups, and the t-test was used to compare 2 groups. For variables that did not pass the normality test, the Kruskal–Wallis with a post hoc Dunn’s was used to compare 3 groups, and the Mann–Whitney test was used to compare 2 groups. The two different VEGF expression patterns were compared by χ^2^. Correlations between continuous variables were evaluated by using the Pearson test or the Spearman test, depending on the variables. The Kaplan–Meier method was used to compare the overall survival of patients depending on the VEGF expression pattern. For the remaining markers, the Cox regression model, adjusted for age, was used for overall survival analyses.

The receiver operating characteristic (ROC) area under the curve (AUC) was used to determine the angiogenic markers’ diagnostic accuracy. All statistical analyses were performed by using the GraphPad Prism (version 8.01), except for the Cox regression analysis, which was performed by using the SPSS software (version 26.00 for Windows). A *p* < 0.05 was considered statistically significant.

## 5. Conclusions

This is the first report to describe the expression of Ang–Tie pathway in ACT. When compared to benign tumors, ACC presented a lower vascular density but a higher Ang2 expression, a biomarker related to vascular permeability and cell spreading. Additionally, Tie1 expression was higher in ACC with venous invasion and pertaining to patients with lower overall survival. Although functional and mechanistic studies are still needed to validate these results, our data support a role for the Ang–Tie pathway in ACT angiogenesis.

## Figures and Tables

**Table 1:** VEGF immunostaining localization in the different groups of adrenocortical tumors.

Groups	*n*	Expression Pattern		
		Nucleus + Cytoplasm	Cytoplasm	*p*
ACC	21	8 (38%)	13 (62%)	0.09
ACAc	8	0 (0%)	8 (100%)	
ACAn	13	5 (38%)	8 (62%)	

ACAc, Adrenocortical adenoma with Cushing syndrome; ACAn, non-functioning adrenocortical adenoma; ACC, adrenocortical carcinoma.

**Table 2:** Percentage of stained area of each angiogenic marker according to subgroup analysis ENSAT score, metastasis, capsular, venous and sinusoids invasion.

		CD34	VEGF-R2	Ang1	Ang2	Tie1	Tie2
ENSAT score	1–2	2.589 ± 0.538	9.676 ± 3.342	15.477 ± 2.278	44.699 ± 5.810	0.106 ± 0.046	0.284 ± 0.282
	3–4	4.728 ± 0.944	15.771 ± 1.767	14.824 ± 1.473	45.449 ± 5.727	0.206 ± 0.111	0.239 ± 0.178
	*p*	0.051	0.138	0.945	>0.999	0.731	>0.999
Metastasis	Yes	3.473 ± 0.533	17.458 ± 1.670	14.880 ± 2.207	53.200 ± 2.804	0.116 ± 0.058	0.094 ± 0.094
	No	3.859 ± 0.891	10.958 ± 2.460	15.235 ± 1.889	41.105 ± 5.294	0.179 ± 0.089	0.333 ± 0.219
	*p*	0.940	0.106	0.940	0.283	0.825	0.471
Capsular Invasion	Yes	3.957 ± 0.710	13.950 ± 1.664	14.030 ± 1.204	42.795 ± 3.700	0.233 ± 0.095	0.193 ± 0.111
	No	2.478 ± 0.548	9.840 ± 4.857	14.585 ± 2.55	45.314 ± 16.004	0.155 ± 0.068	0.565 ± 0.564
	*p*	0.291	0.365	0.945	>0.999	0.734	0.739
Venous Invasion	Yes	3.914 ± 0.628	19.527 ± 2.987	14.099 ± 1.390	41.141 ± 5.186	**0.431 ± 0.133**	0.245 ± 0.162
	No	3.474 ± 0.889	12.871 ± 2.633	14.709 ± 1.704	45.182 ± 3.686	**0.099 ± 0.031**	0.235 ± 0.187
	*p*	0.351	0.166	0.793	0.536	**0.021**	0.872
Sinusoidal Invasion	Yes	4.158 ± 0.761	16.006 ± 2.871	14.245 ± 1.585	43.456 ± 3.733	0.215 ± 0.084	0.335 ± 0.165
	No	2.808 ± 0.289	12.350 ±2.742	14.590 ± 1.639	43.456 ± 8.110	0.193 ± 0.039	0.014 ± 0.011
	*p*	0.411	0.446	0.446	>0.999	0.379	0.850

Ang1, angiopoietin 1; Ang2, angiopoietin 2; ENSAT, European Network for the Study of Adrenal Tumors; Tie1, tyrosine kinase with immunoglobulin-like and EGF-like domain 1; Tie2, tyrosine kinase with immunoglobulin-like and EGF-like domain 2; VEGF-R2, vascular endothelial growth factor receptor 2. Statistically significant differences are highlighted in bold.

**Table 3:** Overall survival in patients with ACC.

Angiogenic Marker	HR	95% CI	*p*
CD34	0.69	0.36–1.33	0.27
VEGF	0.60	0.10–3.46	0.59
VEGF-R2	1.12	0.97–1.30	0.13
Ang1	1.07	0.89–1.28	0.48
Ang2	1.03	0.95–1.12	0.49
Tie1	25.93	0.62–1087.29	0.09
Tie2	1.89	0.42–8.45	0.40

Ang1, angiopoietin 1; Ang2, angiopoietin 2; HR, hazard ratio; Tie1, tyrosine kinase with immunoglobulin-like and EGF-like domain 1; Tie2, tyrosine kinase with immunoglobulin-like and EGF-like domain 2; VEGF, vascular endothelial growth factor; VEGF-R2, vascular endothelial growth factor receptor 2; 95% CI, 95% confidence.

**Table 4:** Patients’ and tumors’ characteristics.

	ACC	ACA	
		N/F	Cushing
N	22	13	8
Age at surgery	54 ± 11	34 ± 8	59 ± 12
Sex F:M	15:7	9:3	7:1
Tumor size (cm) (range)	10 ± 5.6 (2.7–20)	4.1 ± 2.2 (1.8–9.5)	3.5 ± 0.98 (2.4–5)
Weiss score (range)	3–8	0–1	0
ENSAT score		NA	NA
1	15%		
2	31%		
3	31%		
4	23%		
Functionality			
N/F	9%	100%	
Cortisol	9%	0	100%
Aldosterone	5%	0	0
Androgens	9%	0	0
Cortisol + Androgens	5%	0	0
Unknown	63%	0	0

ACA, adrenocortical adenoma; ACC, adrenocortical carcinoma; ENSAT, European Network for the Study of Adrenal Tumors; NA, not applicable; N/F, non-functioning.

**Table 5:** Summary table of positive control, antigen retrieval, washing solution and dilution for each antibody used.

Antibody	CD34 (ab81289)	VEGF (ab52917)	VEGF-R2 (ab2349)	Ang1 (ab8451)	Ang2 (ab153934)	Tie1 (ab201986)	Tie2 (ab24859)
Positive control	kidney	kidney	breast	lung	placenta	kidney	lung
Antigen retrieval	Microwave treatment in 0.01 M citrate buffer at pH 6.0 with 0.05% Tween 20 during 15 min	Pressure-cooking boiling for 3 min in 0.01 M citrate buffer at pH 6.0		Microwave treatment in 0.01 M citrate buffer at pH 6.0 during 15 min		Pressure cooking boiling for 3 min in 0.01 M citrate buffer at pH 6.0 with 0.05% Tween 20	Pressure cooking boiling for 3 min in 0.01 M citrate buffer at pH 6.0 with 0.25% Triton-X
Washing solutions	PBS 0.05% Tween 20	PBS	PBS	PBS	PBS	PBS 0.05% Tween 20	PBS 0.05% Triton-X
Primary antibody dilution	1:2000	1:100	1:100	1:400	1:400	1:100	1:100

Ang1, angiopoietin 1; Ang2, angiopoietin 2; PBS, phosphate buffer saline; Tie1, tyrosine kinase with immunoglobulin-like and EGF-like domain 1; Tie2, tyrosine kinase with immunoglobulin-like and EGF-like domain 2; VEGF, vascular endothelial growth factor; VEGF-R2, vascular endothelial growth factor receptor 2.

## Data Availability

The data presented in this study are available upon request from the corresponding author.

## Supplementary Materials

The following are available online at https://www.mdpi.com/article/10.3390/ijms23105579/s1.
