# Supplementary material for: Ang–Tie Angiogenic Pathway Is Distinctively Expressed in Benign and Malignant Adrenocortical Tumors
Source: Int J Mol Sci. 2022 May 17;23(10):5579. doi: 10.3390/ijms23105579 (PMC9146687; doi:10.3390/ijms23105579)
Supplement: Supplementary file 1 [file ijms-1668920-supplementary.pdf › ijms-1668920-supplementary.pdf]

Ang-Tie angiogenic pathway is distinctively expressed in benign and malignant adrenocortical tumors

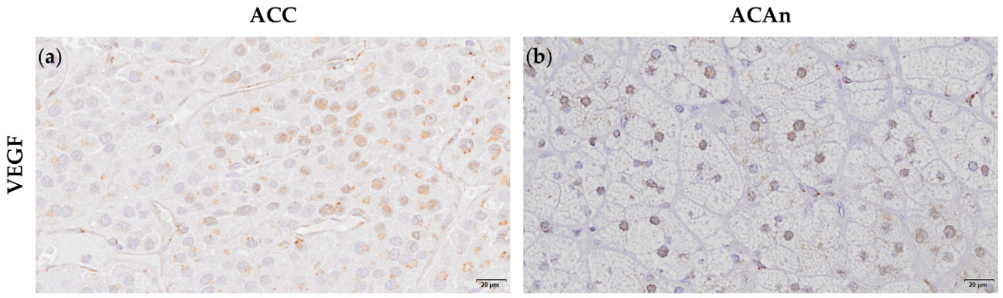

Figure S1. Immunochemistry staining of adrenocortical carcinomas (ACC) (**a**) and non-functioning adrenocortical adenoma (ACAn) (**b**) for VEGF. VEGF nuclear staining in ACC and ACAn (**a**, **b**).

Ang-Tie angiogenic pathway is distinctively expressed in benign and malignant adrenocortical tumors

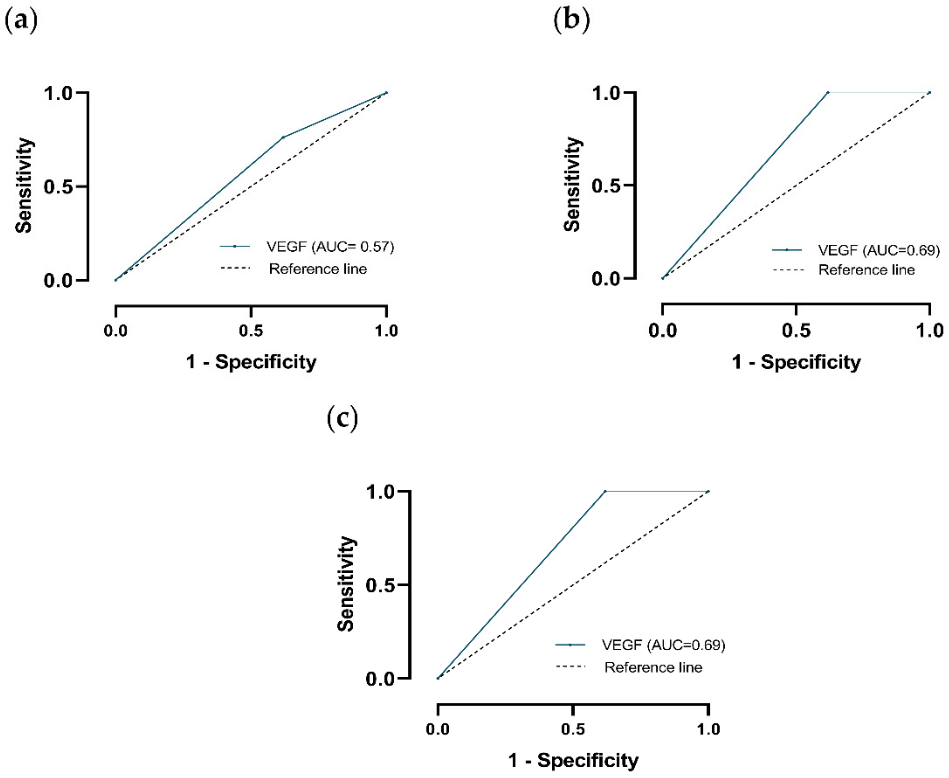

Figure S2. ROC curves to assess the accuracy of VEGF to distinguish between adrenocortical carcinomas (ACC) and total adrenocortical adenomas (ACAt) (**a**); ACC and adrenocortical adenomas with Cushing syndrome (ACAc) (**b**); ACC and non-functioning adrenocortical adenoma (ACAn) (**c**) with the respective area under the curve (AUC).

Ang-Tie angiogenic pathway is distinctively expressed in benign and malignant adrenocortical tumors

Table S1. Correlation between the angiogenic markers and patient/tumor characteristics in ACC.

Correlation	*p*

Angiogenic marker	Patient/ Tumor characteristics	Coefficient correlation (r)	
**CD34**	Age	0.080	ns
	Tumor size	-0.138	ns
	Weiss score	0.006	ns
	ENSAT score	0.285	ns
**VEGF-R2**	Age	-0.188	ns
	Tumor size	0.136	ns
	Weiss score	0.279	ns
	ENSAT score	0.441	ns
**Ang1**	Age	0.376	ns
	Tumor size	0.055	ns
	Weiss score	0.128	ns
	ENSAT score	0.043	ns
**Ang2**	Age	-0.004	ns
	Tumor size	0.317	ns
	Weiss score	0.152	ns
	ENSAT score	0.336	ns
**Tie1**	Age	0.096	ns
	Tumor size	0.092	ns
	Weiss score	0.060	ns
	ENSAT score	0.006	ns
**Tie2**	Age	-0.126	ns
	Tumor size	0.202	ns
	Weiss score	-0.175	ns
	ENSAT score	-0.158	ns

Ang1: Angiopoietin 1; Ang2: Angiopoietin 2; ENSAT: European Network for the Study of Adrenal Tumors; ns: non-significant; Tie1: Tyrosine kinase with immunoglobulin-like and EGF-like domain 1; Tie2: Tyrosine kinase with immunoglobulin-like and EGF-like domain 2; VEGF-R2: Vascular endothelial growth factor receptor 2.

Ang-Tie angiogenic pathway is distinctively expressed in benign and malignant adrenocortical tumors

Table S2. Correlation between the angiogenic markers and patient/tumor characteristics in ACAc.

Correlation		Coefficient correlation (r)	*p*
**Angiogenic marker**	**Patient/ Tumor characteristics**		
**CD34**	Age	-0.538	ns
	Tumor size	0.655	ns
	Weiss score	0.131	ns
**VEGF-R2**	Age	0.357	ns
	Tumor size	-0.509	ns
	Weiss score	0.393	ns
**Ang1**	Age	0.429	ns
	Tumor size	0.436	ns
	Weiss score	0.655	ns
**Ang2**	Age	0.500	ns
	Tumor size	0.127	ns
	Weiss score	0.393	ns
**Tie1**	Age	-0.250	ns
	Tumor size	0.164	ns

	Weiss score	-0.655	ns
**Tie2**	Age	0.143	ns
	Tumor size	0.291	ns
	Weiss score	0.393	ns

Ang1: Angiopoietin 1; Ang2: Angiopoietin 2; ENSAT: European Network for the Study of Adrenal Tumors; ns: non-significant; Tie1: Tyrosine kinase with immunoglobulin-like and EGF-like domain 1; Tie2: Tyrosine kinase with immunoglobulin-like and EGF-like domain 2; VEGF-R2: Vascular endothelial growth factor receptor 2.

Ang-Tie angiogenic pathway is distinctively expressed in benign and malignant adrenocortical tumors

Table S3. Correlation between the angiogenic markers and patient/tumor characteristics in ACAn.

Correlation		Coefficient correlation (r)	*p*
**Angiogenic marker**	**Patient/ Tumor characteristics**		
**CD34**	Age	0.073	ns
	Tumor size	-0471	ns
	Weiss score	-	
**VEGF-R2**	Age	0.093	ns
	Tumor size	0.311	ns
	Weiss score	-	
**Ang1**	Age	0.091	ns
	Tumor size	0.471	ns
	Weiss score	-	-
**Ang2**	Age	0.030	ns
	Tumor size	0.546	ns
	Weiss score	-	
**Tie1**	Age	0.358	ns
	Tumor size	0.101	ns
	Weiss score	-	

Ang1: Angiopoietin 1; Ang2: Angiopoietin 2; ENSAT: European Network for the Study of Adrenal Tumors; ns: non-significant; Tie1: Tyrosine kinase with immunoglobulin-like and EGF-like domain 1; VEGF-R2: Vascular endothelial growth factor receptor 2. Correlation between expression of the angiogenic markers and the Weiss score as not performed, since the score for all ACAn was 0.

Ang-Tie angiogenic pathway is distinctively expressed in benign and malignant adrenocortical tumors

Table S4. Percentage of ACC samples with different VEGF expression localization pattern according to various parameters including ENSAT score, metastasis, capsular, venous and sinusoidal invasion.

		Cytoplasm + Nucleus expression	Cytoplasm expression
**ENSAT score**	1-2	50.00%	50.00%
	3-4	50.00%	50.00%
	p	>0.99	
**Metastasis**	Yes	33.33%	16.67%
	No	66.67%	83.33%
	p	>0.99	
**Capsular Invasion**	Yes	85.71%	71.43%
	No	14.29%	28.57%
	p	>0.99	

**Venous Invasion**	Yes	28.57%	55.56%
	No	71.43%	44.44%
	p	0.36	
**Sinusoidal Invasion**	Yes	57.14%	87.50%
	No	42.86%	12.50%
	p	0.28	

ENSAT: European Network for the Study of Adrenal Tumors.
